# Mild sodium reduction in peritoneal dialysis solution improves hypertension in end stage kidney disease: a case-report study

**DOI:** 10.1186/s12882-021-02380-4

**Published:** 2021-05-08

**Authors:** Luigi Vecchi, Mario Bonomini, Roberto Palumbo, Arduino Arduini, Silvio Borrelli

**Affiliations:** 1 Unit of Nephrology, Santa Maria Hospital, Terni, Italy; 2 Department of Medicine, Section of Nephrology and Dialysis, G. d’Annunzio University, Chieti-Pescara, Chieti, Italy; 3 Unit of Nephrology, Sant’Eugenio Hospital, Rome, Italy; 4 R&D Department, Iperboreal Pharma, Pescara, Italy; 5 Department of Advanced Medical and Surgical Sciences, Nephrology Unit of University of Campania “Luigi Vanvitelli”, Piazza Miraglia, 80138 Naples, Italy

**Keywords:** Low-sodium, Resistant hypertension, Peritoneal Dialysis

## Abstract

**Introduction:**

Blood Pressure (BP) control is largely unsatisfied in End Stage Kidney Disease (ESKD) principally due to sodium retention. Peritoneal Dialysis (PD) is the most common type of home dialysis, using a peritoneal membrane to remove sodium, though sodium removal remains challenging.

**Methods:**

This is a case-study reporting two consecutive ESKD patients treated by a novel peritoneal PD solution with a mildly reduced sodium content (130 mmol/L) to treat hypertension.

**Results:**

In the first case, a 78-year-old woman treated by Continuous Ambulatory PD (CAPD) with standard solution (three 4 h-dwells per day 1.36% glucose 132 mmol/L) showed resistant hypertension confirmed by ambulatory blood pressure monitoring (ABPM), reporting 24 h-BP: 152/81 mmHg, day-BP:151/83 mmHg and night-ABP: 153/75 mmHg, with inversion of the circadian systolic BP rhythm (1.01), despite use of three anti-hypertensives and a diuretic at adequate doses. No sign of hypervolemia was evident. We then switched from standard PD to low-sodium solution in all daily dwells. A six-months low-sodium CAPD enabled us to reduce diurnal (134/75 mmHg) and nocturnal BP (122/67 mmHg), restoring the circadian BP rhythm, with no change in ultrafiltration or residual diuresis. Diet and drug prescription were unmodified too.

The second case was a 61-year-old woman in standard CAPD (three 5 h-dwells per day) suffering from hypertension confirmed by ABPM (mean 24 h-ABP: 139/84 mmHg; mean day-ABP:144/88 mmHg and mean night-ABP:124/70 mmHg). She was switched from 132-Na CAPD to 130-Na CAPD, not changing dialysis schedule. No fluid expansion was evident. During low-sodium CAPD, antihypertensive therapy (amlodipine 10 mg and Olmesartan 20 mg) has been reduced until complete suspension. After 6 months, we repeated ABPM showing a substantial reduction in mean 24 h-ABP (117/69 mmHg), mean diurnal ABP (119/75 mmHg) and mean nocturnal ABP (111/70 mmHg). Ultrafiltration and residual diuresis remained unmodified. No side effects were reported in either cases.

**Conclusions:**

This case-report study suggests that mild low-sodium CAPD might reduce BP in hypertensive ESKD patients.

## Introduction

Blood Pressure (BP) control is largely unsatisfied in End Stage Kidney Disease (ESKD) [1, 2]. Poor BP control is principally due to sodium retention leading to extra-cellular volume (ECV) expansion, which is commonly detectable in ESKD patients [3], significantly worsening the cardio-vascular prognosis of these patients [4]. However, sodium removal remains challenging in dialysis patients.

Peritoneal Dialysis (PD) is the most common type of home dialysis, using a peritoneal membrane to remove sodium and fluid overload [5]. However, in PD sodium removal depends substantially on ultrafiltration, so that an increase in sodium removal needs a high concentration of glucose and/or icodextrin [6]. But hypertonic solutions remove more water than sodium [6] and, furthermore, chronic exposure to glucose load causes local toxicity [7] and metabolic consequences [8]. On the other hand, icodextrin is able to remove sodium, but can only be used once a day with a limited effect on sodium removal by diffusion [9]. Hence, novel strategies are desirable to increase sodium removal and improve BP control.

One therapeutic strategy is lowering the sodium in the PD solution, which may improve sodium removal by diffusion. However, the use of an ultra-low sodium (102–115 mEq/L) dialysate commonly leads to hyponatremia, hypotension and diuresis contraction, while these solutions need to be compensated by a hypertonic glucose solution to avoid ultrafiltration (UF) loss [10–14]. More recently, the use of uncompensated PD solutions with a relatively low sodium content (125 mEq/L) has resulted in an improvement of BP control in PD patients, though there persists a higher risk of hyposodiemia than with a standard PD solution [13, 14].

Our working hypothesis was that a mild, but persistent, reduction in the sodium content of the PD dialysate (130 mmol/L) might help to improve BP control, reducing the sodium load in ESKD patients; moreover, this slight reduction in the PD bag sodium content could be better tolerated.

Hence, we used a novel uncompensated glucose 130 mmol Na PD solution to treat two consecutive hypertensive CAPD patients for 6 months, aiming at evaluate its effect on the BP burden.

## Case presentation

### Patient n.1

A 78-year-old woman suffering from End Stage Kidney Disease showed high BP levels resistant to antihypertensive treatment. Written informed consent was obtained from the patient for publication of this case report.

She had a medical history characterized by at least 10 years of poor BP control, despite use of three anti-hypertensives (doxazosin 4 mg, amlodipine 10 mg, telmisartan 80 mg) and a diuretic (furosemide 250 mg) at the maximum tolerated dose. A low salt diet (< 100 mmol/day) had also been prescribed. Physical examination showed no lower-limb edema or any signs of volume expansion. Ultrasound disclosed that the left kidney was reduced in size, whereas the contralateral kidney showed a high intrarenal resistance index (0.82) suggestive of atherosclerotic reno-vascular disease, with unilateral renal artery stenosis, as the apparent cause of resistant hypertension. The patient refused radiological intervention, so in view of the kidney function deterioration (serum Creatinine: 5.14 mg/dL estimated GFR by CKD-EPI: 6 mL/min/1.73m^2^) and poor BP control, she was put on continuous ambulatory PD (CAPD) using 3 times 2-L exchanges of 1.36% standard PD solution (Dianeal, Baxter® sodium:132 mmol/L) and an empty abdomen during the nocturnal hours.

After 3 months of CAPD, BP levels persisted elevated (mean Home BP: 152/80 mmHg,), despite euvolemia revealed by echocardiography (collapsibility of inferior vena cava). We therefore performed ABPM (SpaceLabs Healthcare®) to exclude pseudo-resistant hypertension [15]. ABPM yelded poor control of mean 24 h-BP (152/81 mmHg), mean diurnal (151/83 mmHg) and mean nocturnal BP (153/75 mmHg), with an inversion of the circadian rhythm (systolic night/day ratio: 1.02). Considering that antihypertensive treatment was still optimal, we switched from standard PD (132 mEq/L) to a low sodium PD solution using 1.4% glucose bags with a sodium concentration of 130 mEq/L (DextroCore LS, Iperboreal Pharma, Italy). The CAPD schedule was confirmed.

As reported in Table 1, over 16 weeks we registered a clinically significant BP reduction (− 7 mmHg) measured by automatic sphygmomanometer (Omron M3®) at home, with no substantial change in body weight, UF, total Kt/V or residual diuresis. The patient was trained to use the Omron BP device by medical personnel according to HBP guidelines [16]. Furthermore, no main lab data were modified, while the diet and therapy prescription were unmodified. Peritoneal equilibration test (PET) showed average peritoneal permeability (dialysate/plasma creatinine ratio:0.76).

**Table 1:** Description of the main clinical and laboratory features at baseline and after 6 months of 130 mmol Na CAPD

	Patient 1		Patient 2	
	Baseline	130-Na CAPD	Baseline	130-Na CAPD
Creatinine (mg/dL)	5.14	5.17	5.28	6.72
Measured GFR (mL/min/1.73 m^2^)	6.0	6.3	9.9	6.2
Urine volume (mL/24 h)	1300	1600	1700	1600
Body weight (kg)	56.0 ± 0.4^a^	56.7 ± 0.3^b^	69.0 ± 0.7^a^	70.3 ± 0.6^b^
Ultrafiltration (mL/24 h)	330 ± 126^a^	365 ± 113^b^	450 ± 135^a^	485 ± 132^b^
Total Ktv	1.65/week	1.68/week	1.75/week	1.80/week
Serum sodium (mmol/L)	137	137	138	139
Serum potassium (mmol/L)	4.9	4.3	4.1	3.8
Serum Urea (mg/dL)	173	177	144	161
Serum Hemoglobin (mg/dL)	11.6	12.4	11.5	10.4
Serum Albumin (g/L)	3.3	3.6	3.9	3.5
Serum PTH (pg/mL)	297	250	589	357
Home Systolic BP (mmHg)	147 ± 7^a^	140 ± 9^b^	135 ± 5^a^	120 ± 3^b^
Home Diastolic BP (mmHg)	78 ± 5^a^	73 ± 5^b^	85 ± 4^a^	70 ± 3^b^

^a^Mean of all available measurements before the switch (*N* = 86); ^b^Mean of all home measurements after the switch (*N* = 115). All BP measurements were performed by automatic sphygmomanometer (Omron M3®). Patients were trained according to EURECA-m, ERA-EDTA and ESH guidelines [16]. Body weight was gauged by automatic scale. Ultrafiltration was calculated by difference in the weight of dialysate before and after each dwell

**Fig. 1:**
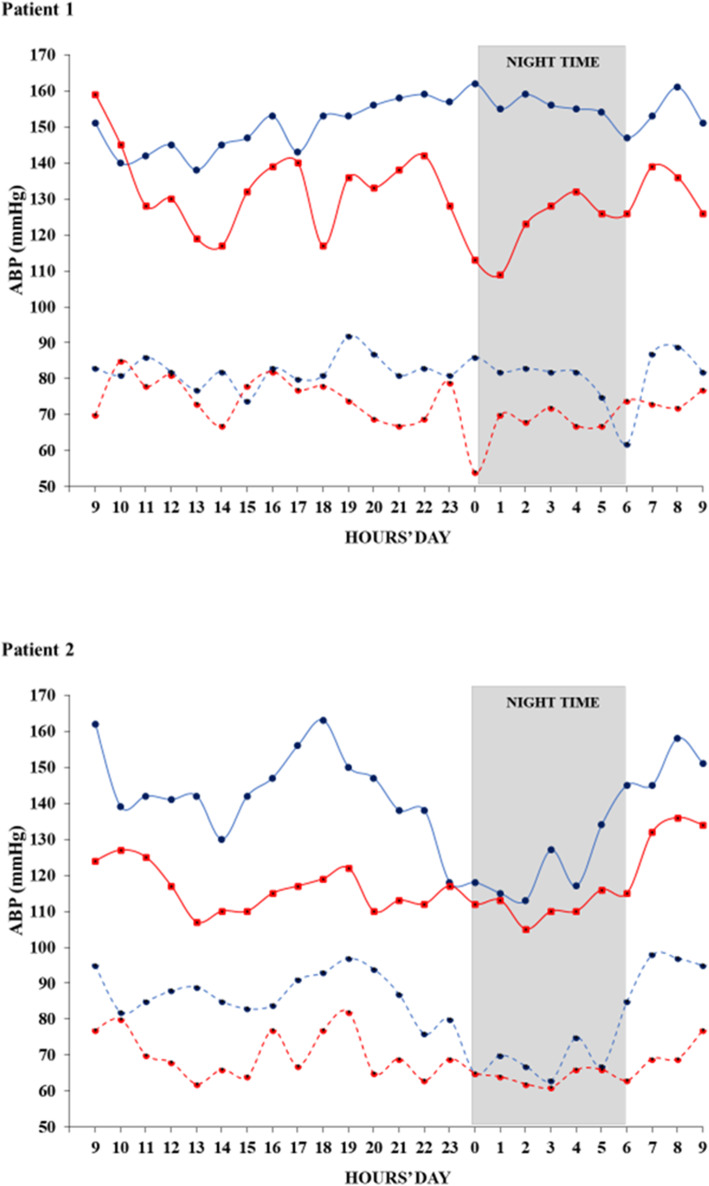
Systolic (solid line) and Diastolic (dotted line) Ambulatory Blood Pressure at baseline (blue) and after 6 months (red) of Na-130 CAPD in two consecutive hypertensive patients

After 6 months we performed a second ABPM with mean 24 h ABP of 131/73 mmHg), mean diurnal of 134/75 mmHg and mean nocturnal ABP of 122/67 mmHg. Notably, the circadian rhythm was restored (systolic night/day ratio: 0.91). Figure 1 (graph at the top named as patient 1) illustrates the difference for each hour between two ABPMs (baseline vs 6 months). Lastly, no side effects were reported.

### Patient n.2

A 69-year-old woman suffering from ESKD started CAPD with three 5-h diurnal dwells with standard PD solution containing 1.36% glucose and 132 mmol in sodium. After 3 months of standard CAPD, we performed ABPM, finding mean 24 h-BP of 152/81 mmHg, mean day-BP of 151/83 mmHg and mean night-ABP of 153/75 mmHg. The patient took calcium-antagonist (amlodipine 10 mg) and angiotensin II receptor blocker (Olmesartan 20 mg), with no need of diuretics, because volume expansion was not evident. Based on the previous clinical case, we switched from standard CAPD to Na 130 mmol CAPD (1.40% glucose Dextro-Core LS - Iperboreal Pharma®), not changing dialysis schedule. During 6 months of Na 130 mmol CAPD, antihypertensive therapy was reduced until the complete suspension, based on BP measurements performed at home [16]. As illustrated in Fig. 1 (graph at the bottom named as patient 2), ABPM showed a substantial reduction in mean 24 h-ABP (117/69 mmHg), mean diurnal ABP (119/75 mmHg) and mean nocturnal ABP (111/70 mmHg). No change in body weight, ultrafiltration or residual diuresis was found, while the main clinical and lab features were unmodified during follow up (Table 1). No side effects were reported. PET showed an average pattern of peritoneal permeability (Dialisate/plasma creatinine ratio:0.72).

Written informed consent was obtained from the patients for publication of this case report.

## Discussion and conclusions

In the first case report, we describe a significant BP reduction associated with chronic use of low-sodium (Na 130 mmol), an uncompensated glucose-based PD solution in a female CAPD patient suffering from resistant hypertension. More specifically, we found a significant decrease in systolic ABP (− 16 mmHg) after 6 months of PD treatment with Na-130 glucose bags in all daily dwells (three per day), with no substantial change in body weight (BW) or anti-hypertensive therapy, suggesting that BP lowering is the consequence of maintenance treatment by Na 130 mmol PD solution. Considering these findings, we used by this low-sodium CAPD solution on a second hypertensive patient with poor BP control. Similarly, we found substantial BP improvement that allowed us to suspend all anti-hypertensive drugs. UF, body weight and residual diuresis remained unmodified too.

These findings are consistent with the results of a recent trial comparing uncompensated Na-125 with standard PD solutions, in which a significant improvement was found in systolic BP (− 17 mmHg) associated with use of low sodium PD solution in the subgroup of patients with lower GFR (< 6 ml/min/1.73m^2^), regardless of ultrafiltration [14]. Unlike that previous study [14], we observed that BP control was independent of residual kidney function (RKF) at the baseline (6.1 ml/min/1.73m^2^ in the first patient vs 9.9 ml/min/1.73 m^2^ in the second patient). Furthermore, we noticed a reduction in RKF (from 9.9 to 6.0 ml/min/1.73 m^2^) in the second patient, although no diuresis contraction was found.

Remarkably, the six-months use of 130 mmol/l Na CAPD bags was associated with ABP control in both patients, in accordance with recent guidelines recommending an average BP < 130/80 mmHg over 24-h monitoring in PD patients [16]. ABPMs confirmed the previous findings achieved by Home BP measured during 6-months’ follow up (Table 1). More interestingly, we found restoration of the circadian BP rhythm associated with chronic use of Na-130 PD solution in the first case, which may be indirect proof that the effect on our patient’s BP rhythm is a direct consequence of sodium lowering throughout the body. For a higher salt intake in salt-sensitive hypertension is associated with an abnormal circadian BP rhythm [17] while sodium correction by low-sodium diet and/or diuretics allows one to improve nocturnal hypertension and restore dipping status [18, 19]. Similarly, in the second case, optimal BP control with no need of anti-hypertensive therapy in a CKD patient may be further proof of the benefit of CAPD treatment for the sodium body content. Likewise, we may postulate that use of a low-sodium dialysate, improves the sodium overload in total sodium tissue, regardless of the fluid overload.

The latest evidence suggests that sodium may play a pivotal role in salt-sensitive hypertension pathogenesis regardless of the fluid overload. According to a recent hypothesis, sodium is stored in the interstitial tissue by a local mechanism regulated by the mononuclear phagocyte system, which acts as an osmoreceptor by expression of a tonicity enhancer binding protein. This transcription factor leads to VEGF-C production which increases sodium clearance on the part of the lymphatic network, reducing sodium in the skin interstitium. Abnormalities of this local system lead to a salt-sensitive increase in BP [5]. This sodium accumulation in interstitial tissues is currently detectable in humans by ^23^Na Magnetic Resonance Imaging. Sodium stored in interstitial tissue is higher in CKD [20] and is associated with greater cardiac mass, regardless of volume status  [21]. Recent findings suggest that sodium stored in the skin is not unmodifiable, being removable by dialysis [22], thus opening new scenarios for BP treatment of PD patients.

The nature of case-report study prevents us from drawing any causal conclusion, although our findings provide further insights into the use of a low-sodium solution to treat hypertension in PD patients, suggesting that a slight, but continuous, reduction in sodium PD solution is able to improve hypertension, and, unlike using an ultra-low Na solution [10–12], requires no need of compensated solutions at a higher glucose concentration while the risk of hyponatremia and diuresis contraction is limited. Further studies are mandatory to investigate whether the BP effect was independent of RKF and whether the loss of RKF may be related to low-Na solution or only the consequence of improved BP control.

Our study has other limitations. First, we did not perform bioimpedance to evaluate fluid volume, since we could not exclude insensible fluid overload. Second, we did not assess sodium balance: although low sodium intake was prescribed in both patients, no information about diet and therapy adherence was available, again, sodium removal was not evaluated.

In conclusion, our case-report study suggests that a slight reduction in the sodium content (130 mEq/L) of the PD solution, compared to standard PD solutions available in commerce (132–134 mEq/L), if delivered at all dwells for 6 months, might be enough to improve BP control, probably due to lowering of sodium overload over time. Mild reduction in the sodium content of the PD bag (130 mmol/L) might be considered as “a right compromise” between the need to reduce the sodium load and avoiding ultrafiltration loss and glucose load. Our findings must necessarily be confirmed by randomized controlled trials aimed at proving the efficacy of such a novel sodium PD solution in hypertensive ESKD patients.

## Data Availability

The information about this study is available contacting corresponding author (SB).

## Abbreviations

ESKD: End stage kidney disease
CAPD: Continuous ambulatory peritoneal dialysis
PD: Peritoneal dialysis
ABPM: Ambulatory blood pressure monitoring
BP: Blood pressure
CKD: Chronic kidney disease
GFR: Glomerular filtration rate
BW: Body weight
UF: Ultrafiltration
VEGF-C: Vascular endothelial growth factor – C
RKF: Residual kidney function
